# Contrasting Social Media Use Between Young Adults With Inflammatory Bowel Disease and Type 1 Diabetes: Cross-sectional Study

**DOI:** 10.2196/34466

**Published:** 2022-04-25

**Authors:** Susruthi Rajanala, Jennifer K Wilson, Paul D Mitchell, Katharine C Garvey, Laurie N Fishman

**Affiliations:** 1 Division of Gastroenterology, Boston Children's Hospital Harvard Medical School Harvard University Boston, MA United States; 2 Institutional Centers for Clinical and Translational Research Boston Children's Hospital Harvard University Boston, MA United States; 3 Division of Endocrinology, Boston Children's Hospital Harvard Medical School Harvard University Boston, MA United States

**Keywords:** social media, inflammatory bowel disease, type 1 diabetes, internet, young adult, children, Instagram, Facebook, type 1, diabetes

## Abstract

**Background:**

Social media is used by young adult patients for social connection and self-identification.

**Objective:**

This study aims to compare the social media habits of young adults with inflammatory bowel disease (IBD) and type 1 diabetes (T1D).

**Methods:**

This is a cross-sectional study of subjects from Boston Children’s Hospital outpatient IBD and diabetes clinics. Patients above 18 years of age were invited to complete a brief anonymous survey, which asked about the various ways they use several social media platforms.

**Results:**

Responses were received from 108 patients (92.5% response rate), evenly split across disease type. We found that 83% of participants spent at least 30 minutes per day on social media, most commonly on Instagram and Facebook. Although the content varied based on the platform, patients with IBD posted or shared content related to their disease significantly less than those with T1D (23% vs 38%, *P*=.02). Among Instagram users, patients with IBD were less likely to engage with support groups (22% vs 56%, *P*=.04). Among Twitter users, patients with IBD were less likely to seek disease information (77% vs 29%, *P*=.005). Among Facebook users, patients with IBD were less likely to post about research and clinical trials (31% vs 65%, *P*=.04) or for information seeking (49% vs 87%, *P*=.003). Patients with IBD were also less likely to share their diagnosis with friends or family in person.

**Conclusions:**

Young adults with IBD were less willing to share their diagnosis and post about or explore the disease on social media compared to those with T1D. This could lead to a sense of isolation and should be further explored.

## Introduction

Inflammatory bowel disease (IBD) and type 1 diabetes (T1D) are both prevalent chronic diseases with significant impact on health and quality of life [1,2]. Young adults in particular are affected by the social and interpersonal impact of these conditions [3] at a time when social interaction with peers is often central. Young adults may face loss of other familiar social structures at this time such as moving away from the family home, starting college, starting a job, and transitioning their medical care to new providers [4,5].

Social media consists of a rapidly changing collection of internet tools and phone apps. However, literature has consistently shown that adolescents and young adults use social media to make social connections, seek support, explore self-identity, and learn self-presentation and disclosure [6]. Prior to the COVID-19 pandemic, 76% of American teenagers used at least one form of social media, with an average daily usage of 1 hour and 11 minutes [6], and this likely increased with the onset of the pandemic. Social media is a commonly used tool that may provide clues about how young adults are feeling through their self-disclosures, discussion of their condition, searches for information, or avoidance of the topic.

Young adults with IBD and T1D are useful comparisons as these conditions vary in public familiarity and stigma. IBD has been reported to have very poor public familiarity and was ranked in one study as having higher social stigma than genital herpes, alcoholism, and obesity [7]. Diabetes is more common in the general population and better recognized. Some studies have evaluated social media use by a cohort of patients with a single condition such as IBD, but there is limited literature about social media patterns of patients with contrasting conditions [8]. Specifically, to our knowledge, no prior study has explored and compared social media usage of adolescents with IBD and those with T1D. This comparison can be used to help begin to tease out which factors influence how young adults use social media with respect to their medical condition. We hypothesize that patients with T1D will know others with the condition and will be more inclined to share health information. In contrast, we wonder if patients with IBD will share less as the condition is less well known and has more associated stigma [9].

## Methods

### Participants and Data Collection

This cross-sectional pilot study included 108 young adult patients who presented to outpatient Boston Children’s Hospital IBD and diabetes clinics from October 2019 to January 2020. Patients were given an envelope with the invitation letter and the survey and could return the envelope with the survey blank or filled out. Of the 126 patients given envelopes, 108 filled in answers. Information for the study was collected in an anonymous paper-based survey. Those who were younger than 18 years old or who were not proficient in English were excluded. Eligible patients were informed about the study and that submission of the anonymous survey would imply consent to use their answers for research.

The survey was created to explore specific issues not found in validated instruments. One round of pilot testing was done with a group of 4 patients. Further iterations were done with providers knowledgeable about surveys and these conditions and young adults with other conditions. The instrument has not yet been validated.

The patient survey collected demographics, including participants’ age, gender, race, diagnosis (Crohn disease or ulcerative colitis, for the IBD group only), time since diagnosis, and self-reported disease severity on a scale of 1-5, with 5 being most severe. The survey explored participants’ social media usage and posting patterns, along with in-person habits such as how often and with whom they discuss their diagnoses.

### Ethics Approval

This study was approved by the Boston Children’s Hospital Institutional Review Board (IRB-P00032571).

### Statistical Analysis

Patient age and disease severity are described with mean (SD) and all other patient characteristics with frequency (percentage). Patient characteristics were compared between the T1D and IBD groups using the standardized difference to assess balance. To do this, propensity scores (*P_i_*) were obtained from a logistic regression model using Firth penalized maximum likelihood estimation to reduce bias in the parameter estimates due to low prevalence of some predictors. A total of 3 indicator variables for disease history (1-2, 3-5, or 6 or more years ago; referencing <1 year ago) and 5 indicator variables for disease severity (1=mild, 5=most severe; referencing unknown severity) were included in the model. Inverse probability of treatment weights (IPTW) were calculated as 1/*P_i_* for the *i^th^* observation in the T1D group, and as 1/(1–*P_i_*) for the *i^th^* observation in the IBD group. Standardized differences were calculated for each patient characteristic *X* as 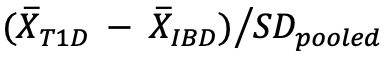, where 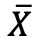 = mean of patient characteristic *X* and SD_pooled_ = standard deviation pooled over the two groups. Absolute standardized differences <0.25 were deemed negligible [10]. The overlap of the distributions of estimated propensity score by disease type (common support) was assessed graphically (Multimedia Appendix 1).

Respondent characteristics (age, sex, race, ethnicity) as well as disease severity and time since diagnosis are described with unweighted summary statistics. Survey questions regarding how much patients thought about their disease and how much they discussed their disease were reverse coded so that higher scale numbers were associated with higher frequency of behavior. Categories of questionnaire (Likert scale) items are reported as weighted (IPTW) percentages and summarized by weighted median and IQR for each disease group. To avoid confusion, the frequencies corresponding to weighted percentages are not shown since the weighting resulted in fractional quantities that were not directly comparable to the observed sample sizes. For categorical (nonordered) survey questions, comparisons between disease groups were made with the Rao-Scott chi-square statistic. For Likert scale items, the nonparametric Jonckheere-Terpstra test for ordered categories was used to compare groups; it has greater power for ordered categories than the Wilcoxon rank-sum test. All comparisons are unadjusted for other covariates with statistical tests (*P* values) based on the propensity score weighted (IPTW) data. All tests of significance were 2-sided, with *P*<.05 considered statistically significant. Analysis was performed with SAS (version 9.4; SAS Institute).

## Results

A total of 108 patients completed the study questionnaire and they were evenly split across disease type. Participants consisted of 59 male patients and 49 female patients, and 11% (12/108) were Hispanic or Latino. Mean age was 20.3 (SD 2.1) years (range 18-25), and median self-reported disease severity was 3 (IQR 2-3; range 1-5, from mild to most severe). The majority of participants (68/108, 63%) were diagnosed more than 5 years ago. Absolute standardized differences were beyond the negligible threshold of >0.25 for age (0.35) and disease severity ratings 1 (mild; 0.57), 2 (0.33), and 3 (0.62). After applying inverse probability of treatment weights, all patient characteristics had absolute standardized differences <0.25 (range 0.00-0.12; Table 1), considered negligible.

**Figure 1:**
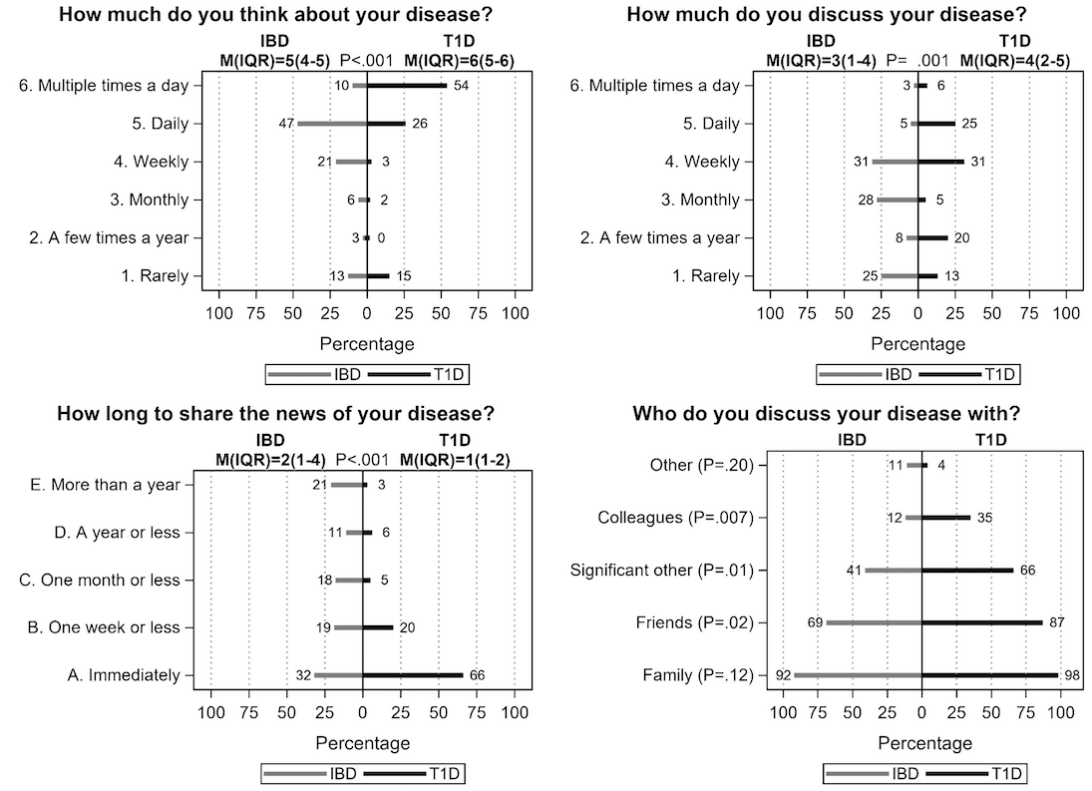
Comparison of in-person interactions by disease type. Weighted percentage and median (IQR) are shown for "select one response" questions with 2-group comparison by Jonckheere-Terpstra test, and weighted percentage for "check all that apply" questions compared with 2-group comparison by Rao-Scott chi-square test. IBD: inflammatory bowel disease; T1D: type 1 diabetes.

**Figure 2:**
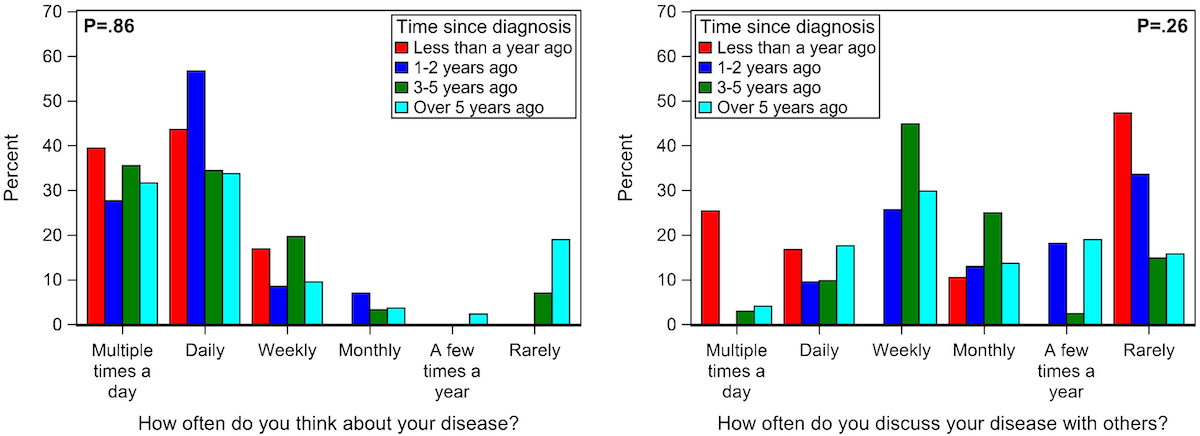
Association of time since disease diagnosis by (A) how often you think about your disease and (B) how often you discussed your disease with others. *P* value from Rao-Scott chi-square test after combining (1) weekly and rarely categories, and (2) <1 year, 1-2 years, and 3-5 years ago, to avoid table cells containing zeros.

Overall, patients with IBD and T1D appear to have different patterns of in-person interactions regarding their diagnoses (Figure 1). Patients with T1D reported thinking about their disease and discussing their disease with others more often when compared to patients with IBD (thinking: median 6, IQR 5-6 vs median 5, IQR 4-5, *P*<.001; discussing: median 4, IQR 2-5 vs median 3, IQR 1-4, *P*<.001). Those with T1D were also quicker (lower score) to share their diagnosis with others than patients with IBD (median 1, IQR 1-2 vs median 2, IQR 1-4, *P*<.001). Compared to patients with IBD, those with T1D were more likely to discuss their disease with friends (87% vs 69%, *P*=.001), their significant other (66% vs 41%, *P*<.001), and colleagues (35% vs 12%, *P*<.001; Figure 1). There was no correlation between time since diagnosis and how often patients thought about (*P*=.86) or discussed their disease with others (*P*=.26; Figure 2). Finally, compared to patients with IBD, those with T1D were more likely to report knowing family/friends (62% vs 38%, *P*=.002) or celebrities with their diagnosis (71% vs 29%, *P*=.01; data not shown).

**Table 1:** Patient characteristics (N=108).

Characteristics			Unweighted									Standardized difference (T1D–IBD)^a,b^		
			All respondents (N=108)		IBD (n=54)		T1D (n=54)		Absolute difference (T1D–IBD)		Unweighted			Weighted
Age (years), mean (SD)			20.3 (2.1)		20.6 (2.2)		19.9 (1.9)		–0.7 (2.1)		–0.35			0.12
**Sex, n (%)**														
	Male	59 (55)		32 (59)		27 (50)		–5 (8)		–0.19			0.08	
	Female	49 (45)		22 (41)		27 (50)		5 (8)		0.19			–0.08	
Hispanic or Latino, n (%)			12 (11)		4 (7)		8 (15)		4 (8)		0.24			0.03
White^c^, n (%)			89 (87)		45 (88)		44 (86)		–1 (2)		–0.05			0.00
**Disease severity^d^, n (%)**														
	1 (mild)	11 (11)		10 (19)		1 (2)		–9 (17)		–0.57			0.08	
	2	30 (29)		19 (37)		11 (21)		–8 (16)		–0.33			–0.05	
	3	46 (45)		15 (29)		31 (61)		16 (32)		0.62			0.02	
	4	12 (12)		7 (13)		5 (10)		–2 (3)		–0.12			–0.10	
	5 (most severe)	4 (4)		1 (2)		3 (6)		2 (4)		0.20			0.06	
**Years since diagnosed, n (%)**														
	<1	6 (6)		3 (6)		3 (6)		0 (0)		Reference			Reference	
	1-2	9 (8)		6 (11)		3 (6)		–3 (5)		–0.20			–0.06	
	3-5	25 (23)		14 (26)		11 (20)		–3 (6)		–0.13			–0.01	
	>5	68 (63)		31 (57)		37 (68)		6 (11)		0.23			0.06	

^a^T1D: type 1 diabetes.

^b^IBD: inflammatory bowel disease, including Crohn disease (n=38), ulcerative colitis (n=15), and indeterminate colitis (n=1).

^c^N=6 (3 IBD, 3 T1D) unknown. Non-White races were African American (n=5), Asian (n=4), Cape Verdean (n=1), Haitian American (n=1), Native American (n=1), and unspecified other (n=1).

^d^N=5 (2 IBD, 3 T1D) declined to answer. Indicator variables were used to assess balance across the groups.

**Figure 3:**
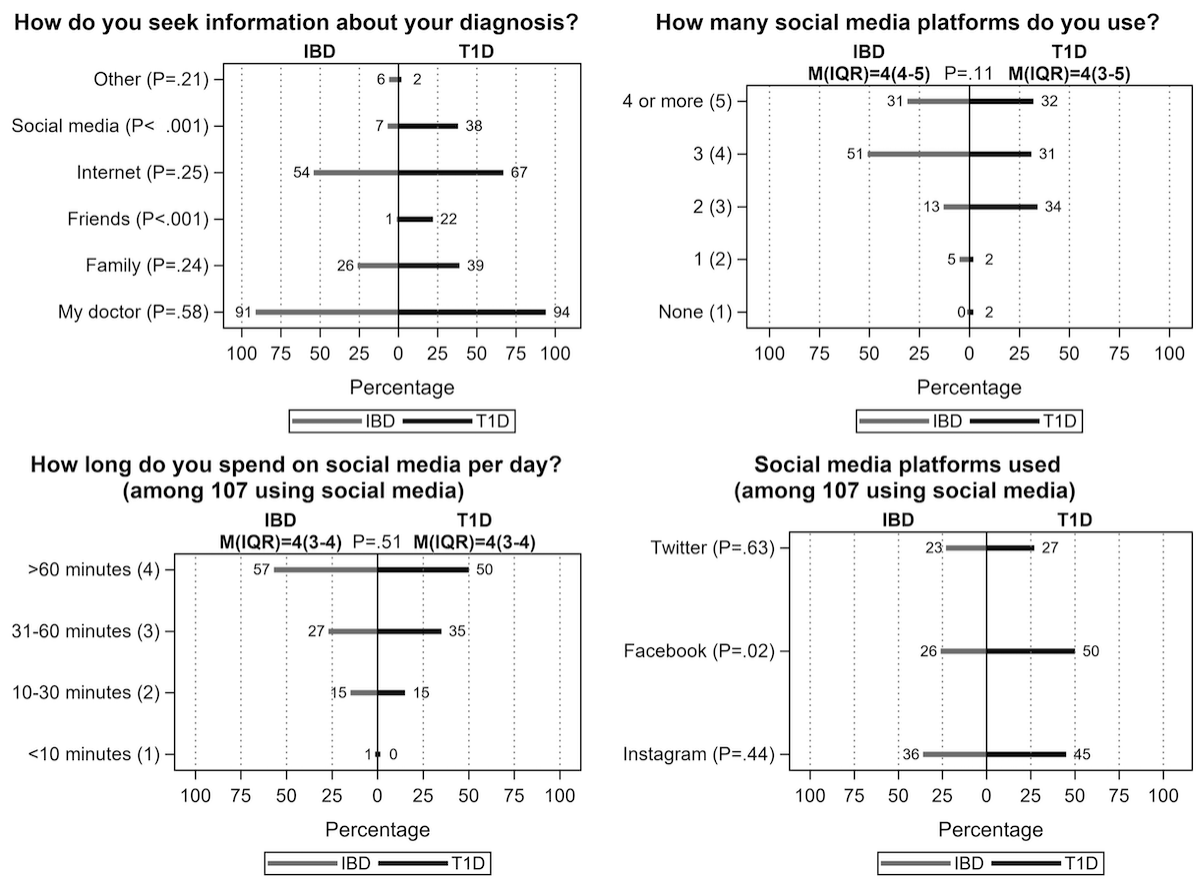
Patient information seeking. Weighted percentage and median (IQR) shown for "select one response" questions with 2-group comparison by Jonckheere-Terpstra test, and weighted percentage for "check all that apply" questions compared with 2-group comparison by Rao-Scott chi-square test. IBD: inflammatory bowel disease; T1D: type 1 diabetes.

**Figure 4:**
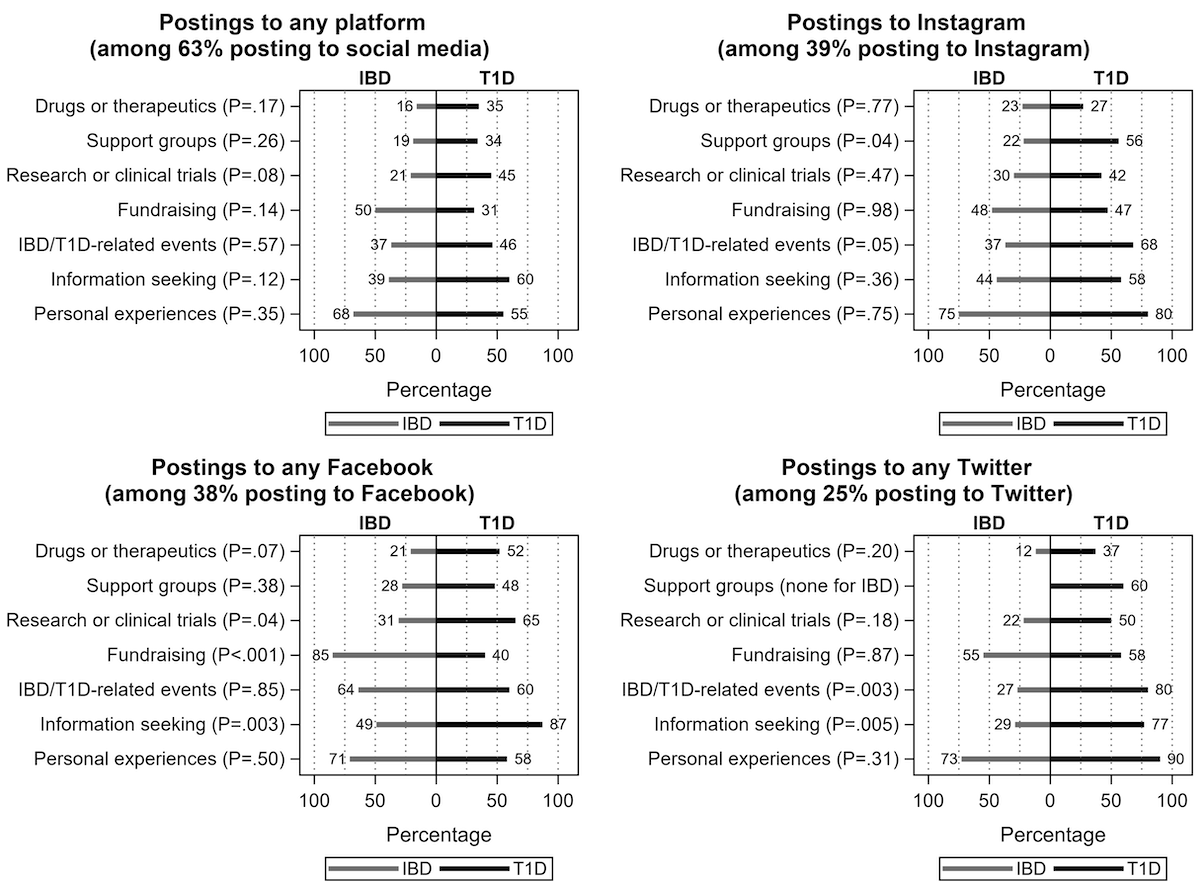
Comparison of information seeking by disease type. Reported are the weighted percentages and Rao-Scott chi-square test results. IBD: inflammatory bowel disease; T1D: type 1 diabetes.

Almost all patients (99%) reported actively using social media, and most (84%) spent at least 30 minutes per day on social media. Instagram was the most common platform (40% of users), followed by Facebook (38%) and Twitter (25%; Figure 3). There was no difference by disease group for amount of time spent on social media; however, Facebook users were more likely to be patients with T1D than IBD (49% vs 26%, *P*=.03; Figure 3). Overall, 73% of patients with T1D vs 51% of patients with IBD (*P*=.03) reported disease-specific social media usage on one or more of these platforms (including searching, reading posts, following other accounts, posting or sharing content) from time of diagnosis until the time of the survey. The most frequent activity related to their personal experiences with their disease (60% posted to at least one platform), ranging from following “new developments or funny moments” or “profiles of others” to posting about one’s disease. The least commonly reported platformwide uses included drugs or therapeutics and research or clinical trials (Figure 4).

Overall, disease-specific social media activity differed by platform. Among Instagram users, patients with T1D were more likely to engage with support groups (56% vs 22%, *P*=.04). Among Twitter users, patients with T1D were more likely to post/share about disease-related events (80% vs 27%, *P*=.003) and for information seeking (77% vs 29%, *P*=.005). Finally, among Facebook users, patients with T1D were more likely to post about research and clinical trials (65% vs 31%, *P*=.04) and for information seeking (87% vs 49%, *P*=.003), while patients with IBD were more likely to post about fundraising (85% vs 40%, *P*<.001; Figure 4). In contrast to high usage rates, only 31% of patients had specifically posted or shared content about their condition across any of these platforms from the time of diagnosis to enrollment date, with 38% of patients with T1D posting or sharing compared to 23% patients with IBD (*P*=.02; data not shown).

## Discussion

### Overview

Our study explores the social media practices of young adult patients with two very different chronic diseases, IBD and T1D. The use of social media was almost universal, and the time spent on social media did not differ by diagnosis. Researchers such as Uhls and Moreno and colleagues [6,11] would argue this is typical for all adolescents and that social media can enable the important developmental tasks of connecting with peers and exploration of identity.

### Disclosing or Sharing

We found that patients with T1D were far more likely than those with IBD (38% vs 23%) to specifically post about their condition on social media. Patients with T1D discussed their diagnosis with others more often and sooner after their diagnosis, with a majority of respondents sharing their diagnosis immediately. Patients with T1D may be more likely to share or discuss their diagnosis as it is more visible in the community and mainstream media, and a feeling of social belonging is important for adolescents [12]. Those with T1D more frequently reported knowing both family/friends and celebrities (eg, singer Nick Jonas) with their disease compared to patients with IBD. In comparison, it has been shown that public familiarity with IBD is poor and comprehension of this disease is limited [7]. Patients with IBD may thus fear that disclosure would not be met with understanding and acceptance. Studies have found that adolescents with IBD preferred not to reveal their condition and cited negative reactions as a major factor [13] and those experiencing stigma had more health communication difficulties. There is a wide range of disclosure by patients.

One Italian study that studied patients with 4 conditions, including IBD and T1D, found that 98% expressed a need to share their medical condition on social media [14]. However, another study of patients with connective tissue disorders noted only 17% revealed their condition on social media [15]. These studies can be used to put the disclosure rate of young adults with IBD and T1D in context.

Those with T1D also thought about their disease more often than patients with IBD, with a majority of participants choosing “multiple times a day,” whereas patients with IBD most often reported “daily” or “weekly.” This is understandable given that patients with diabetes are often on multiple-dose insulin regimens and must carefully consider their diets and adjust dosing for changes such as physical activity or illness [16]. On the other hand, many IBD treatment options are dosed on a biweekly or monthly basis, though some medications must still be taken daily [17].

### Information Gathering

Social media is less commonly used for information gathering than internet sites. Patients with T1D always reported more information gathering than those patients with IBD, although the exact percent varied by platform. This discrepancy is echoed in the literature. One study of the adolescent diabetes population suggested growing interest in using social media to find information [18]. In contrast, a study found that youth with IBD were rarely using social media as an information source [19]. This represents a new avenue for physicians to engage with their patients in an accessible manner. It is also important to educate patients to be critical of the accuracy and quality of health-related information posted on social media, especially if it is used to inform health or treatment-based decisions.

### Community or Connection

Social media can be a powerful tool for creating friendships and connections, particularly among those with similar experiences. We found that, overall, 34% of patients with T1D used social media for support groups compared with 19% of patients with IBD. It is difficult to directly compare one study to another in the literature as the platforms included in the term social media keep shifting. Facebook is one social media platform that has been popular for support groups, particularly for those with rare or embarrassing conditions. The use of private groups on Facebook helps assuage some concerns about privacy. A large number of patients used Facebook to search for friends with the same disease or community support groups to find others who were going through the same thing and could understand their feelings [14]. These online social connections might be particularly important for patients with IBD as some researchers have postulated that the embarrassment of frequent bathroom trips or diarrhea might lead to perceived stigma and social withdrawal [12]. One program used Instagram to augment the social supports for adolescents with T1D to avoid the barriers of travel and time required to attend in person [20]. Online diabetes communities have been shown to be important for peer support as well as problem solving [21].

Another common theme of social media posts for both groups was humor, especially on Twitter. An analysis of humor in the chronic care setting showed patient-initiated humor was most commonly used to deal with negative emotions [22]. Therefore, including this sentiment in social media posts may be an important coping strategy for young adults. Overall, despite low disease-specific posting and sharing rates, young adults in both groups engage with social media in a variety of ways. These platforms can still be an important tool for understanding how young adults feel about and cope with their chronic disease, and may also represent an avenue for providers to interact with their patients.

There are some limitations to this study. This is a single-center study and thus the patient population may not be generalizable. We sought to decrease selection bias by inviting every eligible patient in a consecutive manner and offering a nonconfrontational way to refuse participation, by turning in a black survey inside the envelope. However, selection bias is always present. Young adult responses may be affected by embarrassment or social desirability. The survey instrument also did not ask specifically about Snapchat or TikTok (though some participants did mention Snapchat in the written portion of the survey), which are also popular among this age group. These platforms typically encourage more spontaneous posting or usage, and could represent an important contrast to the other platforms investigated in our study [23,24]. In addition, young adults may have multiple accounts on a single social media platform—for instance, auxiliary accounts on Instagram are colloquially referred to as “finsta,” a portmanteau of “fake” and “Instagram.” These accounts are often less curated and again consist of more spontaneous posting and could also be a key tool for patients to share about or cope with their disease [25]. TikTok has become much more popular even in the time since the study was conducted, and the absence of this platform does limit more current assessment of social media use. Lastly, this study aims to compare social media usage among only two specific patient populations; therefore, it would be advantageous for future research to investigate this topic across institutions and among diverse illnesses.

### Conclusion

Overall, this study expands our understanding of social media use among young adults with chronic disease. To our knowledge, there is limited understanding of how specific chronic conditions impact the use of social media. This study hints at familiarity of disease and stigma around a condition as factors that affect engagement. The more that is known about how patients use these various forms of social media, the more impact providers can have. Patients with IBD seem to communicate far less about their disease compared to patients with T1D in almost all realms across various social media platforms, which has significant implications for education, sense of community, and self-acceptance. Future research is needed for deeper explorations of even more media platforms and a wide array of chronic conditions.

## Appendix

Multimedia Appendix 1 Supplemental figure.

## Abbreviations

IBD: inflammatory bowel disease
IPTW: inverse probability of treatment weights
T1D: type 1 diabetes
