# Supplementary material for: Contrasting Social Media Use Between Young Adults With Inflammatory Bowel Disease and Type 1 Diabetes: Cross-sectional Study
Source: JMIR Pediatr Parent. 2022 Apr 25;5(2):e34466. doi: 10.2196/34466 (PMC9086877; doi:10.2196/34466)
Supplement: Multimedia Appendix 1 [file pediatrics_v5i2e34466_app1.docx]

Supplemental Figure 1. Common support showing the degree of overlap in propensity scores across disease types.
